# Degenerate Near-Planar 3D Reconstruction from Two Overlapped Images for Road Defects Detection

**DOI:** 10.3390/s20061640

**Published:** 2020-03-15

**Authors:** Yazhe Hu, Tomonari Furukawa

**Affiliations:** 1 Department of Mechanical Engineering, Virginia Tech, Blacksburg, VA 24060, USA; 2 Department of Mechanical and Aerospace Engineering, University of Virginia, Charlottesville, VA 22903, USA; tf4rp@virginia.edu

**Keywords:** road surface 3D reconstruction, degenerate reconstruction, road defects detection, pothole detection

## Abstract

This paper presents a technique to reconstruct a three-dimensional (3D) road surface from two overlapped images for road defects detection using a downward-facing camera. Since some road defects, such as potholes, are characterized by 3D geometry, the proposed technique reconstructs road surfaces from the overlapped images prior to defect detection. The uniqueness of the proposed technique lies in the use of near-planar characteristics of road surfaces‘ in the 3D reconstruction process, which solves the degenerate road surface reconstruction problem. The reconstructed road surfaces thus result from the richer information. Therefore, the proposed technique detects road surface defects based on the accuracy-enhanced 3D reconstruction. Parametric studies were first performed in a simulated environment to analyze the 3D reconstruction error affected by different variables and show that the reconstruction errors caused by the camera’s image noise, orientation, and vertical movement are so small that they do not affect the road defects detection. Detailed accuracy analysis then shows that the mean and standard deviation of the errors are less than 0.6 mm and 1 mm through real road surface images. Finally, on-road tests demonstrate the effectiveness of the proposed technique in identifying road defects while having over 94% in precision, accuracy, and recall rate.

## 1. Introduction

A road is one of the most fundamental infrastructures in the transportation system. A healthy and intact road surface condition increases ride comfort and vehicle safety for through traffic [1,2]. The road surface condition inevitably downgrades and is affected by stresses from traffic as well as climate impacts such as humidity or temperature change. Thus, frequent inspections of the road surface are vital in identifying road surface defects along with carrying out timely maintenance. Labor intensiveness, inefficiency, and subjectivity of manual inspection have resultantly necessitated automatic measurement of the road surface defects such as potholes and ruts, which are mostly characterized by geometry [3,4,5,6,7,8].

Past works on automatic road defects detection can be classified into three types: the acceleration-based detection, the color-based detection, and the geometry-based detection. The acceleration-based technique uses accelerometers as irregular geometrical changes create vibration that can be measured by accelerometers. Yu et al. [9] analyzed acceleration and automatically detected road defects for the first time to the best of the authors’ knowledge. Vittorio et al. [10] detected the road anomalies based on the abnormal accelerometer data from the cellphone. Tai et al. [11] and Eriksson et al. [12] proposed a technique using a machine learning approach to detect road anomaly where Support Vector Machine (SVM) and unsupervised learning were used respectively to enhance detection accuracy. Xue et al. [13] adopted a self-learning one degree-of-freedom vibration signal to predict potholes. Mednis et al. [14] implemented and compared several acceleration data processing algorithms for pothole detection, which resulted in a detection rate between 68% to 90%. Although detection by acceleration techniques directly and thus accurately sense geometrical road defects, they miss the detection if no tire steps exactly on the road defects.

For color-based techniques, image sensors are often equipped to obtain the appearance of defects. Tedeschi et al. [15] proposed a technique using Local Binary Pattern (LBP) feature-based cascade classifiers to detect road defects from images. Koch et al. [16,17] used the histogram and four different image filters to extract road distress texture features. Jo et al. [18] constrained the road defect region between two lanes through the lane detection technique to increase the precision of pothole detection. Banharnsakun et al. [19] deployed an Artificial Neural Network (ANN) which can categorize the distress into longitudinal crack, transversal crack, and pothole. Ryu et al. [20] separated the pothole region from the background by Histogram Shape-Based Thresholding (HST) and then used multiple filters to find the pothole features. The color-based technique provides intuitive information about road defects’ position and size. However, the RGB image analysis may not capture geometry and contains unnecessary information such as shadows, oil stains and pavement markings which affect the detection.

Among geometry-based techniques, Chang et al. [21] and Yu et al. [22,23] detected potholes by analyzing topological features obtained from 3D laser scanning data. Hou et al. [24], Fan et al. [25], and El et al. [26] applied stereo-vision systems to extract a 3D point cloud from road surface images and detect potholes directly from the 3D model of the road obtained from point cloud data while no 3D reconstruction precision was investigated. Ahmed et al. [27] proposed a pothole detection technique by Structure from Motion (SfM) taking multiple images on one road surface region to reconstruct 3D points of road surface. While accuracy in depth was reported to be in the order of 0.1 mm, the accuracy was attained by manually marking artificial features on the road surface. Antol et al. [28] and Moazzam et al. [29] implemented the road distress detection by 3D point cloud data from an RGB-D camera. The former used a movable RGB-D camera box to enable depth measurement at a low speed, while the latter mounted on a tripod to statically measure the 3D road surface by the RGB-D camera. However, the accuracy of the 3D reconstruction by using a laser sensor or stereo-vision system can be degraded if the vibration of the measuring sensors is significant. Further, the issue of the 3D reconstruction based technique is its accuracy in 3D reconstruction since the road surface is near-planar and thus provides poor vertical information.

This paper presents a new geometry-based technique that reconstructs road surfaces from two overlapped images captured by a downward-facing camera with little influence caused by the vibration and then detects road defects based on the 3D reconstructed road. The 3D reconstruction performed by using an improved SfM technique is extensively formulated such that the road surfaces, which are near-planar and have small vertical variations, can be reconstructed accurately. By solving the degenerate issue for near-planar road surface reconstruction, the proposed technique thus detects road defects from the accuracy-enhanced 3D reconstructed road surfaces.

This paper is organized as follows. The following section refers to the traditional SfM for the road surface and the degeneracy issue for the planar object reconstruction. Section 3 first presents the proposed 3D reconstruction technique for near-planar road surfaces and then describes the detection of road defects detection based on reconstructed 3D road. Section 4 investigates the ability of the proposed technique parametrically in simulated environments and then applies to real road surface images. Conclusions are summarized in the last section.

## 2. 3D Road Surface Reconstruction from Two Overlapped Images

### 2.1. Problem Formulation

**Figure 1:**
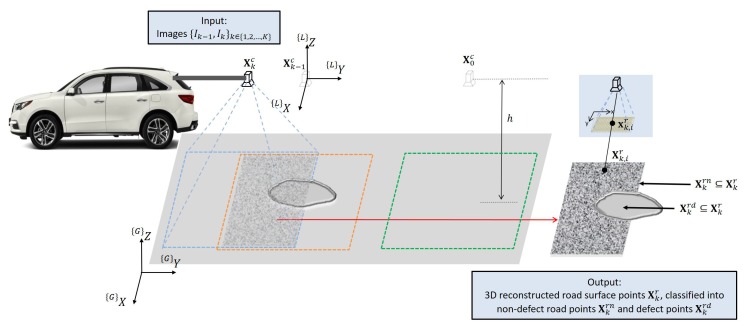
Road surface reconstruction settings for defects detection from one downward-facing camera. 3D point cloud are reconstructed from consecutive images to represent the road surface, followed by classifying the road into defective and non-defective surfaces.

Figure 1 shows general settings and problem formulation of road surface reconstruction using a downward-facing camera for road defects detection. The road surface, shown as a near-planar object, contains a pothole representing a defect road. A camera, facing downward to the road surface at a height *h*, is mounted on a vehicle. While the vehicle is moving, the camera captures images $I_{0:K}$ at positions $X_{0:K}^{c}$ from time step 0 to time step *K*. Since images are captured by a camera of various frame rates at various vehicle speeds, minimally and most fundamentally required is the reconstruction of a 3D road surface overlapped by two consecutive images $\left\{I_{k-1},I_{k}\right\}$. This problem is converted into localizing the road surface point cloud $X_{k}^{r}\equiv \left\{X_{k,i}^{r}|\forall i\right\}$ using the homogeneous two-dimensional (2D) image features $x_{k-1}^{r}\equiv \left\{x_{k-1,i}^{r}|\forall i\right\}$ and the corresponding $x_{k}^{r}\equiv \left\{x_{k,i}^{r}|\forall i\right\}$, which are extracted from image $I_{k-1}$ and $I_{k}$ respectively. It is to be noted that $X_{k}^{c}$ should be derived simultaneously with $X_{k}^{r}$ since the camera position is not precisely known due to the vehicle vibration. Once the reconstruction has been completed, road surface points are classified as normal flat road surface $X_{k}^{rn}$ and defect road surface $X_{k}^{rd}$. In Figure 1, {*G*} represents the global coordinate system while $\left\{L\right\}$ is the local coordinate for two neighboring camera positions.

**Figure 2:**
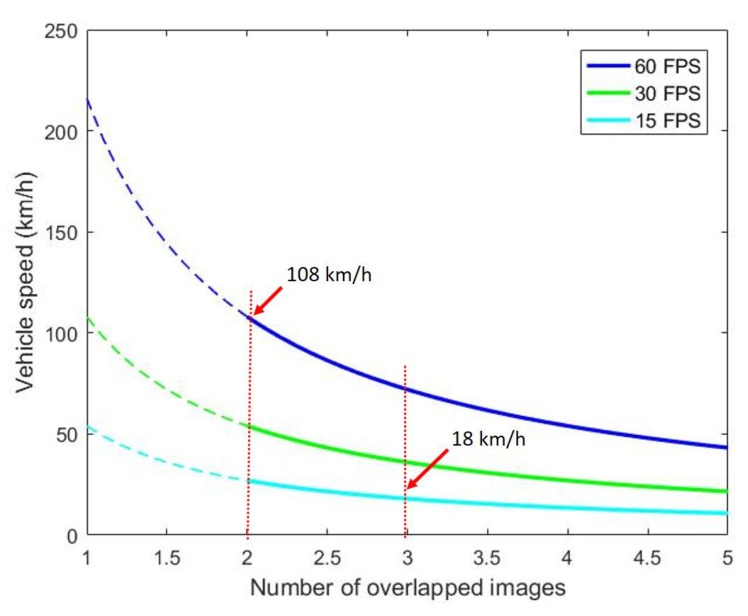
The number of images overlapped on each road surface at various vehicle speed.

Figure 2 illustrates the significance of the two-image problem formulation where the vehicle speed is shown with respect to different numbers of overlapped images when the camera frame rate is 60, 30 and 15 FPS. Note that these are the common frame rates in industrial cameras, and each image covers a 1 m × 1 m road surface area. For every number of images overlapped, $N_{o}$, the overlapping area between every two neighboring images is at least $\left(100-\frac{100}{N_{o}}\right)$%. As the curves exhibit, every camera sees common vehicle speeds when the number of overlapped images is only two. Therefore, 3D road surface reconstruction will fail if it is not possible from two images.

### 2.2. Two-image 3D Road Surface Reconstruction

**Figure 3:**
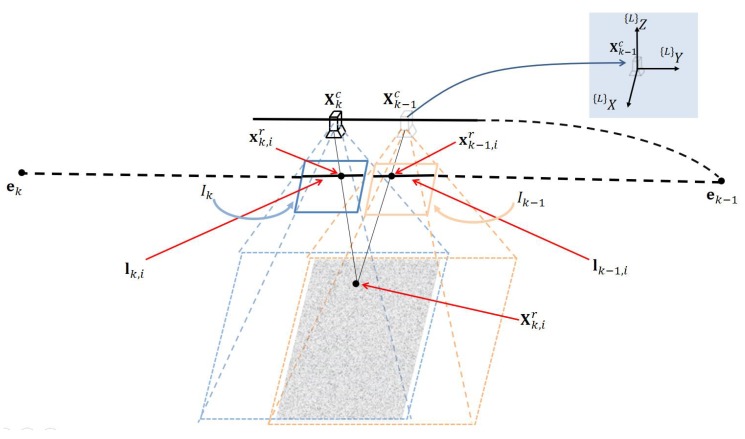
3D road surface reconstruction from two views

Figure 3 shows the notations and the operation of the general road surface 3D reconstruction from image features $x_{k-1}^{r}$ and $x_{k}^{r}$. To present the mathematical derivation of the two-image 3D reconstruction for road surfaces, a line is plotted passing the camera centers, $X_{k-1}^{c}$ and $X_{k}^{c}$. This line intersects with image $I_{k-1}$ at point $e_{k-1}$ as well as image $I_{k}$ at point $e_{k}$. $l_{k-1,i}$ is a line passing through $e_{k-1}$ and $x_{k-1,i}^{r}$, a projection from road surface point $X_{k-1,i}^{r}$ to $I_{k-1}$. Similarly, $l_{k,i}$ is a line passing through $e_{k}$ and $x_{k,i}^{r}$, and this is given by:

(1) $$l_{k,i}=e_{k}\times x_{k,i}^{r}=\left[e_{k}\right]\times x_{k,i}^{r}$$

Combining Equation (1) with ${x_{k,i}^{r}}^{T}l_{k,i}=0$ yields:

(2) $${x_{k,i}^{r}}^{T}\left[e_{k}\right]\times x_{k,i}^{r}=0$$

If $X_{k,i}^{r}$ is located on a road surface plane, then $x_{k-1,i}^{r}$ and $x_{k,i}^{r}$ are related by a homography matrix $H_{ab}$:

(3) $$x_{k,i}^{r}\propto H_{ab}x_{k-1,i}^{r}$$

Substituting Equation (2) to Equation (3)results in:

(4) $${x_{k,i}^{r}}^{T}\left[e_{k}\right]\times H_{ab}x_{k-1,i}^{r}={x_{k,i}^{r}}^{T}F_{k}x_{k-1,i}^{r}=0$$

where $F_{k}=\left[e_{k}\right]\times H_{ab}$ is the fundamental matrix of the two images. Equation (4) holds for all the *n* correspondences $\left\{\left\{x_{k,i}^{r},x_{k-1,i}^{r}\right\}|i=1,2,\ldots ,n\right\}$ [30], which means:

(5) $$\begin{array}{c} {x_{k}^{r}}^{T}F_{k}x_{k-1}^{r}=\\ {\left[\begin{array}{cc} x_{k,1}^{r} & \ldots \;x_{k,n}^{r}\\ y_{k,1}^{r} & \ldots \;y_{k,n}^{r}\\ 1 & \ldots \;1 \end{array}\right]}^{T}\left[\begin{array}{ccc} f_{11} & f_{12} & f_{13}\\ f_{21} & f_{22} & f_{23}\\ f_{31} & f_{32} & f_{33} \end{array}\right]\left[\begin{array}{cc} x_{k-1,1}^{r} & \ldots \;x_{k-1,n}^{r}\\ y_{k-1,1}^{r} & \ldots \;y_{k-1,n}^{r}\\ 1 & \ldots \;1 \end{array}\right]\\ =0 \end{array}$$

The solving of fundamental matrix $F_{k}$, as well as the rotation matrix $R_{k}$ and the translation $t_{k}$ are given by the Appendix A. The final 3D reconstructed road surface $X_{k}^{r}$ is given by the triangulation $f_{t}\left(\cdot \right)$:

(6) $$X_{k}^{r}=f_{t}\left(K,R_{k},t_{k},x_{k}^{r},x_{k-1}^{r}\right)$$

where K is the camera’s intrinsic matrix.

### 2.3. Planar Surface Degeneracy Problem

Since the road surfaces are near-planar, it suffers from the degenerate issue which will be shown by the the rest of this section. As $X_{k}^{r}$ are located on the near-planar road surface, $x_{k-1}^{r}$ and $x_{k}^{r}$ can be related by a 3×3 homography matrix $H_{k}$:

(7) $$x_{k}^{r}\propto H_{k}x_{k-1}^{r}$$

in which $x_{k}^{r}$ is proportional to $H_{k}x_{k-1}^{r}$. This means that the cross product of $x_{k}^{r}$ and $H_{k}x_{k-1}^{r}$ is $x_{k}^{r}\times H_{k}x_{k-1}^{r}=0$. Thus solving $H_{k}$ equals to solving the equation $A^{\prime }h_{k}=0$ where $H_{k}$, $A^{\prime }$ and $h_{k}$ are expressed as:

(8) $$\begin{array}{c} H_{k}=\left[\begin{array}{ccc} h_{11} & h_{12} & h_{13}\\ h_{21} & h_{22} & h_{23}\\ h_{31} & h_{32} & h_{33} \end{array}\right]\\ A_{i}^{\prime }=\left[\begin{array}{cc} -x_{k-1,i}^{r} & 0\\ -y_{k-1,i}^{r} & 0\\ -1 & 0\\ 0 & -x_{k-1,i}^{r}\\ 0 & -y_{k-1,i}^{r}\\ 0 & -1\\ x_{k,i}^{r}x_{k-1,i}^{r} & y_{k,i}^{r}x_{k-1,i}^{r}\\ x_{k,i}^{r}y_{k-1,i}^{r} & y_{k,i}^{r}y_{k-1,i}^{r}\\ x_{k,i}^{r} & y_{k,i}^{r} \end{array}\right],A^{\prime }=\left[\begin{array}{c} A_{1}^{T}\\ A_{2}^{T}\\ .\\ .\\ .\\ A_{n}^{T} \end{array}\right]\\ h_{k}={\left(h_{11},h_{12},h_{13},h_{21},h_{22},h_{23},h_{31},h_{32},h_{33}\right)}^{T} \end{array}$$

To solve $h_{k}$, the problem is equivalent to minimizing $∥A^{\prime }h_{k}∥$ subject to $∥h_{k}∥=1$ because of image noises. Therefore, solving $h_{k}$ is similar to solving $f_{k}$ in the previous section.

Degeneracy is defined as the situation when fundamental matrix $F_{k}$ obtained from the previous procedure is not unique. The planar object, which the road can be approximated as, is one of the degenerate geometries. If $X_{k}^{r}$ are located on a plane surface, the correspondences in the two views $x_{k-1}^{r}$ and $x_{k}^{r}$ satisfy Equation (7). Also, $x_{k-1}$ and $x_{k}$ satisfy Equation (5). The substitution of Equation (7) into Equation (5) yields

(9) $${x_{k}^{r}}^{T}S_{k}x_{k}^{r}=0$$

where $S_{k}=F_{k}H_{k}^{-1}$. To satisfy Equation (9), $S_{k}$ must be a skew-symmetric matrix given by

(10) $$S_{k}=\left[\begin{array}{ccc} 0 & -s_{3} & s_{2}\\ s_{3} & 0 & -s_{1}\\ -s_{2} & s_{1} & 0 \end{array}\right]$$

As a result, the fundamental matrix $F_{k}$ is:

(11) $$F_{k}=S_{k}H_{k}=\left[\begin{array}{ccc} 0 & -s_{3} & s_{2}\\ s_{3} & 0 & -s_{1}\\ -s_{2} & s_{1} & 0 \end{array}\right]H_{k}$$

Thus $F_{k}$ has a solution with three degree-of-freedom (determined by $s_{1},s_{2},$ and $s_{3}$). Since $F_{k}$ is up-to-scale, the solution of $F_{k}$ becomes to have two degree-of-freedom. Therefore the existing 3D reconstruction technique from $I_{k-1}$ and $I_{k}$ cannot lead to correct 3D reconstructed points for planar road surface because of the ambiguity of $F_{k}$ introduced to reconstruction process from Equations (A4) to (A7) and 6. While 3D reconstruction techniques exist, the issue of their direct application to road surface profiling is the ill-posedness of the problem due to the lack of depth information and the incorrect feature matching due to the noisy image. The next section will present the proposed technique, which solves the ambiguity issue of $F_{k}$ for the road surface reconstruction, and leads to correct defects detection based on the 3D information.

## 3. Proposed Degenerate Near-Planar 3D Reconstruction for Road Defects Detection

### 3.1. Overview

**Figure 4:**
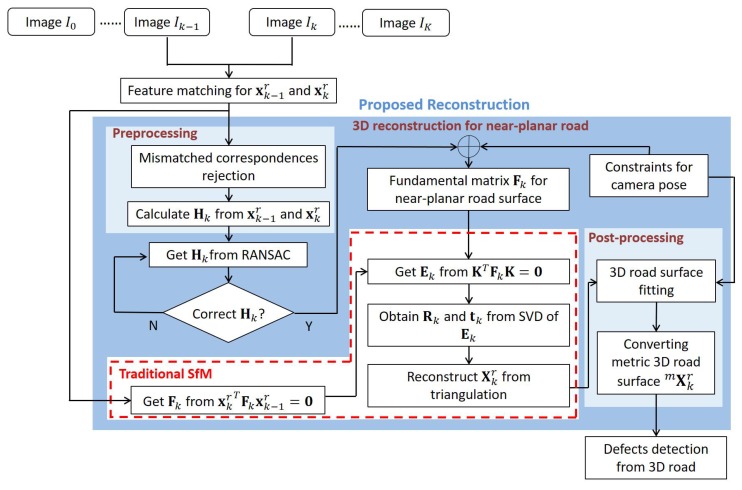
Proposed degenerate near-planar surface reconstruction technique for road defects detection.

Figure 4 shows the proposed degenerate near-planar 3D reconstruction technique for road defects detection. The proposed technique consists of three parts: preprocessing, 3D reconstruction for near-planar road, and post-processing. The preprocessing rejects the mismatched feature correspondences to dramatically improve the feature matching between $I_{k-1}$ and $I_{k}$, which contributes to resolving the degenerate issue for near-planar road surface reconstruction. Then, a newly derived fundamental matrix $F_{k}$ with no ambiguity improves SfM and significantly resolves the degenerate issue. In the post-processing, since the reconstructed points $X_{k}^{r}$ are unitless, the proposed technique converts $X_{k}^{r}$ to metric points ^*m*^$X_{k}^{r}$. As a result, road defects can be detected reliably due to the enhanced accuracy in 3D surface reconstruction.

### 3.2. Preprocessing

The preprocess rejecting mismatched correspondences is formulated as follows. Let the difference of the *i*th corresponding feature at time step k−1 and *k* be:

(12) $$d_{k,i}^{f}\equiv x_{k,i}^{r}-x_{k-1,i}^{r}$$

This makes the set $d_{k}^{f}\equiv \left\{d_{k,i}^{f}|i=1,2,\ldots ,n\right\}$, which includes all the *n* correspondences of the images $I_{k-1}$ and $I_{k}$. As the vehicle is moving along the road following a smooth path, it is valid to assume that the rotation of the camera is small and the camera’s motion is linear in a short period between two neighboring time steps k−1 and *k*:

(13) $$d_{k}^{f}\propto X_{k}^{c}-X_{k-1}^{c}$$

which means $d_{k}^{f}$ are also linear and proportional to the camera’s motion.

Since $I_{k-1}$ and $I_{k}$ has Gaussian noises for $x_{k-1}^{r}$ and $x_{k}^{r}$ and *n* is large with the difference distributed smoothly, the measured image corresponding features ${\hat{x}}_{k-1}^{r}$ and ${\hat{x}}_{k}^{r}$ are:

(14) $${\hat{x}}_{k-1}^{r}=x_{k-1}^{r}+\omega_{k-1},\;\;\;\omega_{k-1}∼N\left(0,\Sigma_{k-1}\right)$$

(15) $${\hat{x}}_{k}^{r}=x_{k}^{r}+\omega_{k},\;\;\;\omega_{k}∼N\left(0,\Sigma_{k}\right)$$

Combining Equations (14) and (15) with Equation (12), the proposed technique models $d_{k}^{f}$ as a Gaussian distribution $d_{k}^{f}∼N\left({\bar{d}}_{k}^{f},\Sigma^{f}\right)$:

(16) $${\bar{d}}_{k}^{f}={\bar{x}}_{k}^{r}-{\bar{x}}_{k-1}^{r},\;\;\;\Sigma^{f}=\Sigma_{k-1}+\Sigma_{k}$$

where ${\bar{d}}_{k}^{f}$ is the mean value and $\Sigma^{f}$ is the covariance matrix of $d_{k}^{f}$. As $d_{k,i}^{f}$ of correct matches are closer to ${\bar{d}}_{k}^{f}$ than those of the mismatched features, mismatched correspondences can be rejected by defining correct matching as:

(17) $$\begin{array}{c} d_{k}^{f,c}=\left\{d_{k}^{f}|{\bar{d}}_{k}^{f}-\lambda \Sigma^{f}1<d_{k}^{f}<{\bar{d}}_{k}^{f}+\lambda \Sigma^{f}1\right\} \end{array}$$

where λ is a threshold and 1 is an all-ones vector. As the exact distance that the camera moves between time step k−1 and *k* is unknown, The RANSAC technique is difficult to determine the threshold and number of iterations to filter correct feature matchings. However, the proposed technique uses the camera’s linear motion as a prior knowledge, which means correct matchings have similar values in $d_{k,i}^{f}$. Unlike RANSAC, Equation (17) only needs to find a reasonable λ and operate once to keep the correct matching within a range $\left({\bar{d}}_{k}^{f}-\lambda \Sigma^{f}1,{\bar{d}}_{k}^{f}+\lambda \Sigma^{f}1\right)$. Therefore, the proposed technique obtains correct feature matchings for the following near-planar 3D reconstruction.

### 3.3. 3D Reconstruction for Near-Planar Road Surface

The proposed technique solves the ambiguity issue of $F_{k}$ by mathematically deriving a unique fundamental matrix for the near-planar road surface. In the local coordinate {*L*}, ^{*L*}^$X_{k-1}^{c}={\left(0,0,0\right)}^{T}$ and its projection to image $I_{k}$, $e_{k}$, is expressed as:

(18) ek=K[Rk,tk]·Xk−1c{L}1=K[Rk,tk]·0001=Ktk

It is noted that from Equation (1), all the lines $l_{k}$ have the following for road surface images:

(19) $$l_{k}=e_{k}\times x_{k}^{r}$$

Meanwhile, Equation (5) and $x_{k}^{rT}l_{k}=0$ relates $F_{k}$ and $l_{k}$ as:

(20) $$F_{k}x_{k-1}^{r}=l_{k}$$

Substitute Equations (19) and (7) into Equation (20) resulting in:

(21) $$F_{k}x_{k-1}^{r}=e_{k}\times x_{k}^{r}=\left[e_{k}\right]\times H_{k}x_{k-1}^{r}$$

Combining Equation (21) with Equation (18), it derives $F_{k}$ for the near-planar road surface as:

(22) $$F_{k}=\left[e_{k}\right]\times H_{k}=\left[Kt_{k}\right]\times H_{k}$$

where $H_{k}$ is calculated recursively by RANSAC using $x_{k-1}^{r}$ and $x_{k}^{r}$ after mismatched points rejection.

Comparing Equation (11) with Equation (22), instead of representing $F_{k}$ with any 3-vector **s**, $F_{k}$ is determined in Equation (22) by $t_{k}$ which is the up-to-scale translation between the camera positions in two views:

(23) $$t_{k}=X_{k}^{c}-X_{k-1}^{c}$$

Since the vehicle moving along the road has small rotation $R_{k}$ for the camera in such a short period from time step k−1 to *k*, $R_{k}$ is expressed as $R_{k}\approx I$. Equation (25) can be obtained from Equation (24):

(24) $$\begin{array}{c} x_{k-1}^{r}=P_{k-1}X_{k}^{r}=K\left[I,0\right]X_{k}^{r}\\ x_{k}^{r}=P_{k}X_{k}^{r}=K\left[R_{k},t_{k}\right]X_{k}^{r}\\ x_{k}^{r}-x_{k-1}^{r}=\left(P_{k}-P_{k-1}\right)X_{k}^{r}=K\left[\left(R_{k}-I\right)\;|\;\left(t_{k}-0\right)\right]X_{k}^{r} \end{array}$$

(25) $$x_{k}^{r}-x_{k-1}^{r}=K\left[0_{3\times 3}\;|\;t_{k}\right]\left[\begin{array}{c} X_{k}^{r}\\ Y_{k}^{r}\\ Z_{k}^{r}\\ 1 \end{array}\right]=Kt_{k}$$

The substitution of Equation (25) into Equation (22) determines $F_{k}$ as:

(26) $$F_{k}={\left[x_{k}^{r}-x_{k-1}^{r}\right]}_{\times }H_{k}$$

As a result, a unique fundamental matrix $F_{k}$ is obtained from Equation (26) when the road surface is near-planar. Then by using the traditional SfM technique, this $F_{k}$ leads to the correct reconstructed road surface points $X_{k}^{r}$ following by identifying defects.

Because of various uncertainties in the 3D reconstruction process, errors will propagate and affect the 3D points $X_{k}^{r}$. Let ${\hat{x}}_{k}^{r}$ be the measured value of $x_{k}^{r}$ where ${\hat{x}}_{k}^{r}=x_{k}^{r}+\omega$ and $\omega ∼N(0,\Sigma_{x_{k}^{r}})$ follows a normal distribution. Equation (24)can be rewritten as:

(27) $$X_{k}^{r}=P_{k}^{+}{\hat{x}}_{k}^{r}$$

where $P_{k}^{+}={\left(P_{k}^{T}P_{k}\right)}^{-1}P_{k}^{T}$ is the pseudo-inverse matrix of $P_{k}$. Let Equation (23) be written as $X_{k}^{r}=f\left(x_{k}^{r}\right)$. By using the first-order Taylor series expansion Equation (23) becomes:

(28) $$f\approx f_{0}+J_{k}{\hat{x}}_{k}^{r}$$

where $J_{k}$ represents the Jacobian matrix of f(·). The covariance matrix of $X_{k}^{r}$ thus is approximated by

(29) $$\Sigma_{X_{k}^{r}}\approx J_{k}\Sigma_{x_{k}^{r}}J_{k}^{T}$$

Since $J_{k}$ in this scenario equals to $P^{+}$ Equation (25) is deduced to be

(30) $$\Sigma_{X_{k}^{r}}\approx P^{+}\Sigma_{x_{k}^{r}}P^{+T}$$

Therefore, although with a unique F for the near-planar road surface, the noises in the image inevitably cause errors for the 3D reconstructed surface points $X_{k}^{r}$ due to the ill-posedness of the problem.

### 3.4. Post-Processing

After getting the near-planar road surface $F_{k}$ with no ambiguity from Equation (26), $X_{k}^{r}$ are reconstructed from Equations (A4) to (A7) and 6. Although, the obtained 3D road surface points $X_{k}^{r}$ are unitless up to a scale factor. In order to get Xkrm, the proposed technique fits a plane on $X_{k}^{r}$ to represent the road surface:

(31) $$\left[\begin{array}{ccc} X_{k}^{r} & Y_{k}^{r} & 1 \end{array}\right]\left[\begin{array}{c} p_{0}\\ p_{1}\\ p_{2} \end{array}\right]=Z_{k}^{r}$$

Then the surface normal vector $n_{k}$ and the up-to-scale distance from the camera to the road surface $h_{u}$ are obtained from $X_{k}^{r}$ based on plane parameters $p_{0},p_{1},$ and $p_{2}$:

(32) $$n_{k}=\frac{\left(p_{0},p_{1},-1\right)}{\sqrt{p_{0}^{2}+p_{1}^{2}+1}}$$

(33) $$h_{u}=\frac{|p_{2}|}{\sqrt{p_{0}^{2}+p_{1}^{2}+1}}$$

The reconstructed surface and the distance $h_{u}$ obtained by Equations (32) and (33), however, may not be the final reconstruction. Because the road surface may have anomalies such as potholes, the first-time road surface reconstruction will be distorted if such anomaly exists. Thus, a recursive surface fitting process is proposed to reconstruct the road surface through Equation (34) to Equation (36):

(34) $$d_{k,i}=\frac{p_{0}X_{k,i}^{r}+p_{1}Y_{k,i}^{r}-Z_{k,i}^{r}+p_{2}}{\sqrt{p_{0}^{2}+p_{1}^{2}+1}}$$

(35) $$X_{k,i}^{r}\in \left\{\begin{array}{c} X_{k}^{rd},if\;d_{k,i}<0\;and\;d_{k,i}\leq T_{d}\\ X_{k}^{rn},else \end{array}\right.$$

(36) $$T_{n}=\frac{size(X_{k}^{rn})}{size(X_{k}^{rn}+X_{k}^{rd})}$$

In Equation (34), $d_{k,i}$ is a signed value calculated as the distance of $X_{k,i}^{r}$ to the current reconstructed road surface. The positive $d_{k,i}$ represents the point $X_{k,i}^{r}$ located in between the camera and the current fitted road surface. The negative $d_{k,i}$ means the point $X_{k,i}^{r}$ is at the other side of the current road surface. Equation (35) illustrates the classification of $X_{k,i}^{r}$ into possible defect points $X_{k}^{rd}$ and non-defect points $X_{k}^{rn}$ by a depth threshold $T_{d}$. $T_{n}$ in Equation (36) is a threshold refers to the percentage of non-defect points among all the points $X_{k}^{r}$. If it is assumed that at least *m* percent of the points $X_{k}^{r}$ are actually representing non-defect road surface, then a $T_{n}>m$ will continue the recursive process to fit a new road surface based on all the $X_{k}^{rn}$ from the last iteration. The recursive process will continue until $T_{n}<m$ is reached.

After the recursive process, an updated camera to road up-to-scale distance $h_{u}$ was obtained from Equation (33). Then a metric scale factor $\alpha_{k}$ is calculated based on the real camera to road surface distance *h*:

(37) Xkrm=αkXkr=hhuXkr

where Xkrm are the metric points with units. From here, the proposed technique converts the up-to-scale points $X_{k}^{r}$ into metric scale road surface points Xkrm. Thus the road defects are detected by the depth (^{*G*}^Z direction) values of Xkrm based on the correct geometry. It is noted here that in order to simplify the notation, Xkrm are still written as $X_{k}^{r}$ in this paper.

## 4. Experimental Results

This section provided two types of experiment to analyze the proposed technique. The first type of experiment was in a Matlab simulated environment which contained the simulated road surface, simulated camera model, and simulated camera motion. The simulation experiments analyzed the influence of different variables to the proposed road surface reconstruction. The second type of experiment was performed on the real road surfaces captured by a road surface imaging system. The real-world experiments demonstrated the accuracy of the proposed technique and its effectiveness on road defects detection.

### 4.1. Experiments in Simulation Environment

**Figure 5:**
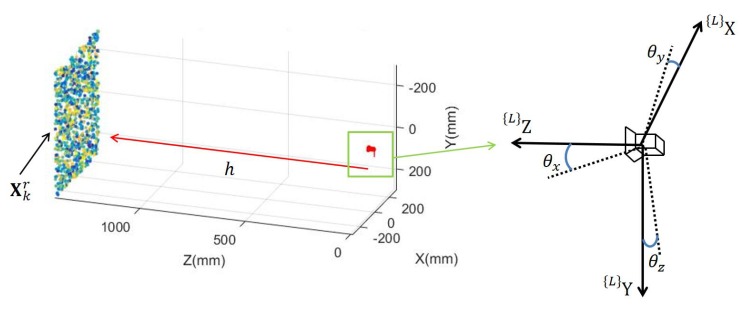
Camera and road surface in the simulation environment.

Figure 5 illustrates the simulated camera and the road surface in the simulation environment. On the right, the simulated camera is facing towards the simulated road surface, and has simulated properties such as intrinsic matrix and field of view. On the left, the environment creates 3D points $X_{k}^{r}\equiv \left\{{\left(X_{k,i}^{r},Y_{k,i}^{r},Z_{k,i}^{r}\right)}^{T}|\forall i\right\}$ to represent the road surface. $Z_{k}^{r}=Z_{m}+\omega_{r}$, where $\;\omega_{r}∼N\left(0,\delta \right)$ is used to change the evenness of the road in ^{*L*}^Z direction. $Z_{m}$ is the mean distance between camera and the road surface. The default unit in the simulation environment is millimeter.

The simulated images are obtained by reprojecting $X_{k}^{r}$ to the simulated camera. ${\hat{x}}_{k}^{r}$ are the measured value of $x_{k}^{r}$ defined as ${\hat{x}}_{k}^{r}=x_{k}^{r}+\omega$, where $\omega ∼N(0,\Sigma_{x_{k}^{r}})$ has the covariance matrix $\Sigma_{x_{k}^{r}}$ and is used to model the uncertainty for matched features in image. The covariance matrix of $\Sigma_{x_{k}^{r}}$ is:

(38) $$\Sigma_{x}=\left[\begin{array}{cc} \sigma^{2} & 0\\ 0 & \sigma^{2} \end{array}\right]$$

As for the orientation, $\theta_{x},\theta_{y}$, and $\theta_{z},$ are the change of angles for the camera about ^{*L*}^X axis, ^{*L*}^Y axis, and ^{*L*}^Z axis between two time steps. Disturbances such as the vibration of the camera cause the orientation change of the camera. Define the error for 3D reconstruction as

(39) $$\epsilon =\frac{1}{N}\sum_{i=1}^{N}|{\hat{d}}_{k,i}/d_{k}^{c}-d_{k,i}/Z_{m}|\cdot Z_{m}$$

where ${\hat{d}}_{k,i}$ is the measured distance and Equation (34) shows the ground truth distance $d_{k,i}$. Table 1 lists the experimental parameters analyzed in the experiment.

**Figure 6:**
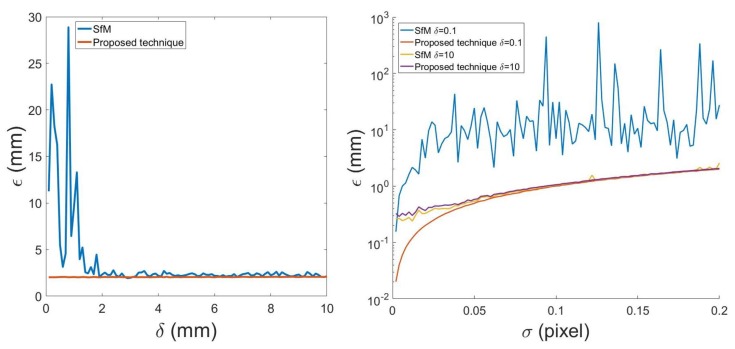
Left: 3D reconstruction error comparison between the proposed technique and traditional SfM when road unevenness δ is changing from 0 to 10 mm. Right: 3D reconstruction error comparison between the proposed technique and traditional SfM at different image noise σ while δ=0.1 or 10 mm.

Figure 6 shows the comparison of 3D reconstruction error between the proposed technique and traditional SfM. The left figure shows the 3D reconstruction error when the road surface is changing from planar (δ=0) to non-planar (δ>>0). When δ is small, the reconstruction error is large for traditional SfM as the degenerate issue still exists, while the proposed technique has small reconstruction errors. The error for the proposed technique in this case is mainly from image noise σ. When the road surface is non-planar, both SfM and the proposed technique have reconstruction error ϵ≈2 mm. The right figure shows the reconstruction error influenced by image noise σ at δ=0.1 and δ=10. For non-planar road surface which has δ=10 mm, the proposed technique and traditional SfM both have small and similar reconstruction error. When δ=0.1 mm, i.e., road surface is near-planar, SfM has error usually between 10 and 1000 mm while the proposed technique has error usually less than 1 mm, and even for a much worse case when σ=0.2, the error is less than 2 mm.

**Figure 7:**
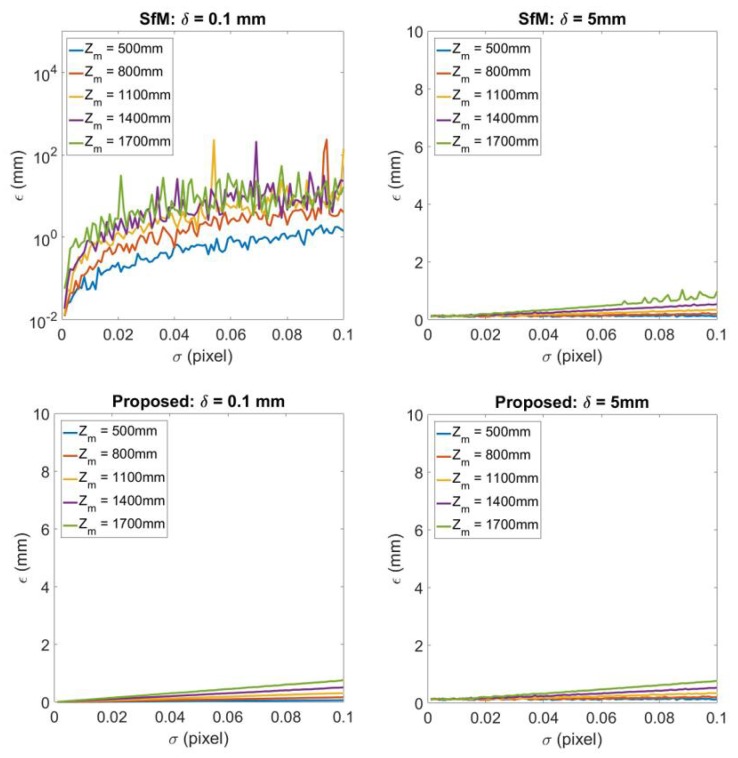
3D reconstruction error for different image noise σ=0.001,0.002,…,0.1. From left to right each column represents the results for road unevenness δ=0.1 and δ=5 respectively. For each column, the top figure shows the 3D reconstruction by traditional SfM technique, while the bottom figure illustrates 3D reconstruction by the proposed technique.

Figure 7 demonstrates the comparison between the traditional SfM and proposed reconstruction technique for planar road and non-planar road 3D reconstruction with different σ. The columns from left to right illustrate the 3D reconstruction under δ=0.1 and δ=5 respectively. For each column, the top figure is the 3D reconstruction error obtained by traditional SfM and the bottom figure is the error of 3D reconstruction by the proposed technique. The image uncertainty σ is changed from 0.001 to 0.1, while the experiment also alters the distance from camera to road surface $Z_{m}$ to discover the influence to the results. It can be discovered that when δ becomes larger which means the road is not a planar surface, SfM gives close results to the proposed technique. When δ becomes smaller the error for SfM increases but for the proposed technique the error remains small.

**Figure 8:**
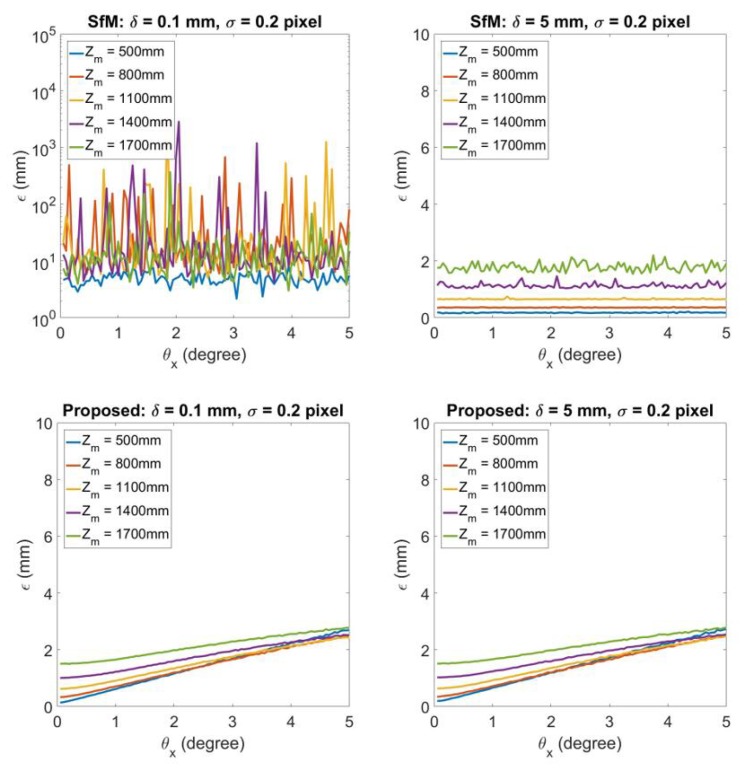
3D reconstruction error for $\theta_{x}=0.05{}^{\circ},0.1{}^{\circ},\ldots ,5{}^{\circ}$. From left to right each column represents the results for δ=0.1,5 respectively. For each column, the top figure shows the 3D reconstruction by SfM, while the bottom figure illustrates 3D reconstruction by the proposed degenerate reconstruction technique.

**Figure 9:**
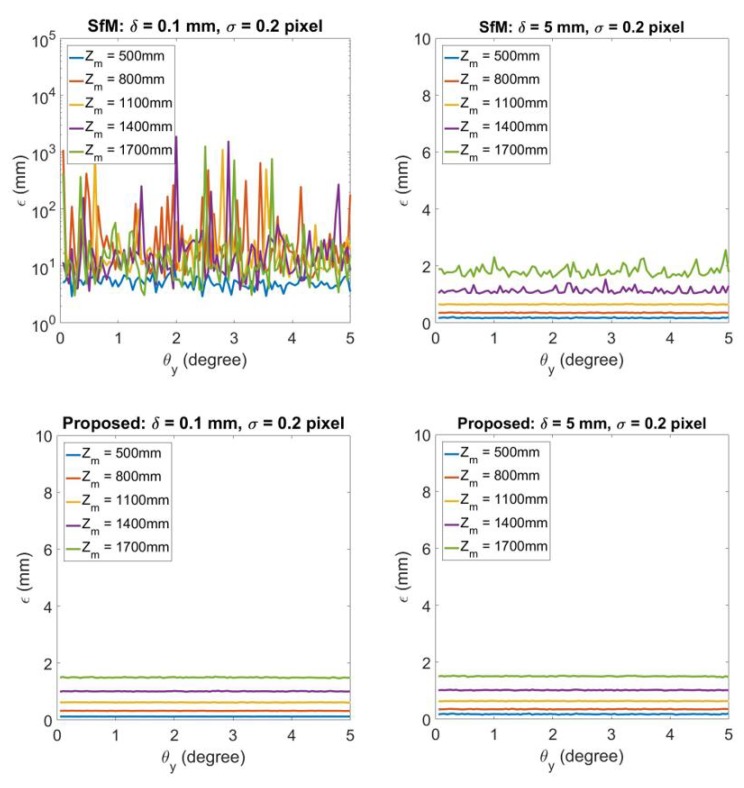
3D reconstruction error for $\theta_{y}=0.05{}^{\circ},0.1{}^{\circ},\ldots ,5{}^{\circ}$. From left to right each column represents the results for δ=0.1,5 respectively. For each column, the top figure shows the 3D reconstruction by SfM, while the bottom figure illustrates 3D reconstruction by the proposed degenerate reconstruction technique.

**Figure 10:**
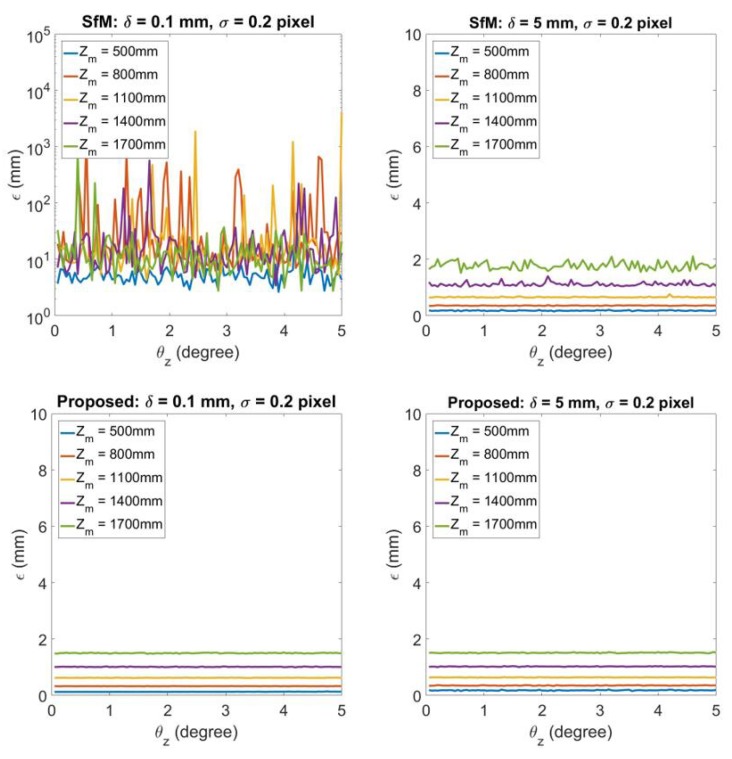
3D reconstruction error for $\theta_{z}=0.05{}^{\circ},0.1{}^{\circ},\ldots ,5{}^{\circ}$. From left to right each column represents the results for δ=0.1,5 respectively. For each column, the top figure shows the 3D reconstruction by SfM, while the bottom figure illustrates 3D reconstruction by the proposed degenerate reconstruction technique.

Figure 8, Figure 9 and Figure 10 shows the 3D reconstruction error ϵ by the influence of errors in rotation matrix **R**. In this simulation experiment, the rotation matrix **R** is decomposed as $R=R_{z}R_{y}R_{x}$ where

(40) $$\begin{array}{c} R_{x}=\left[\begin{array}{ccc} 1 & 0 & 0\\ 0 & \cos\theta_{x} & -\sin\theta_{x}\\ 0 & \sin\theta_{x} & \cos\theta_{x} \end{array}\right]\\ R_{y}=\left[\begin{array}{ccc} \cos\theta_{y} & 0 & \sin\theta_{y}\\ 0 & 1 & 0\\ -\sin\theta_{y} & 0 & \cos\theta_{y} \end{array}\right]\\ R_{z}=\left[\begin{array}{ccc} \cos\theta_{z} & -\sin\theta_{z} & 0\\ \sin\theta_{z} & \cos\theta_{z} & 0\\ 0 & 0 & 1 \end{array}\right] \end{array}$$

$R_{x},R_{y},R_{z}$ are the rotation matrices about the ^*L*^X axis, ^*L*^Y axis, and ^*L*^Z axis correspondingly. The initial camera pose has $\theta_{x}=0{}^{\circ},\theta_{y}=0{}^{\circ},$ and $\theta_{z}=0{}^{\circ}$. $t={\left(200,30,0\right)}^{T}$ in this simulation experiment. In Figure 8, it demonstrates the 3D reconstruction error by changing $\theta_{x}$. From left to right, each column represents the result under δ=0.1,5. σ is set to be 0.2 to represent a worse (relatively large) image noise. The top figure in each column illustrates the results of using traditional SfM, while bottom figure represents the results using the proposed technique. Figure 9 and Figure 10 represents the same experiment by changing $\theta_{y}$ and $\theta_{z}$.

Figure 8 shows the influence to 3D reconstruction error by different $\theta_{x}$. For the SfM results, when δ=0.1 the error is usually more than 5% of camera-to-road distance because in this case the error is dominated by the influence of the degenerate issue. In the meantime, 3D reconstruction error is much less by using the proposed technique for the planar road surface. For δ=5 SfM has error under 2 mm. While for the proposed technique, when $\theta_{x}=5{}^{\circ}$, the error is only around 1 mm larger than the 3D reconstruction error using SfM.

Figure 9 identifies the influence to 3D reconstruction error by different $\theta_{y}$. The error is large and dominated by the influence of degenerate issue for SfM when δ=0.1, while the proposed technique constructs road with less than 2 mm error. When δ=5, SfM has comparable error with the proposed technique. For the proposed, the change of $\theta_{y}$ has little influence on the 3D reconstruction errors which are under 2 mm even at the worst case.

Figure 10 demonstrates the influence to 3D reconstruction error by different $\theta_{z}$. For δ=0.1, the error is also large for traditional SfM because of the degenerate issue while the error is small for the proposed technique. When δ=5, traditional SfM has comparable error with the proposed technique. For the proposed, the change of $\theta_{z}$ almost has no influence to the 3D reconstruction error. The error in this case is mainly influenced by the variable $Z_{m}$. The larger the $Z_{m}$, the larger the error ϵ.

**Figure 11:**
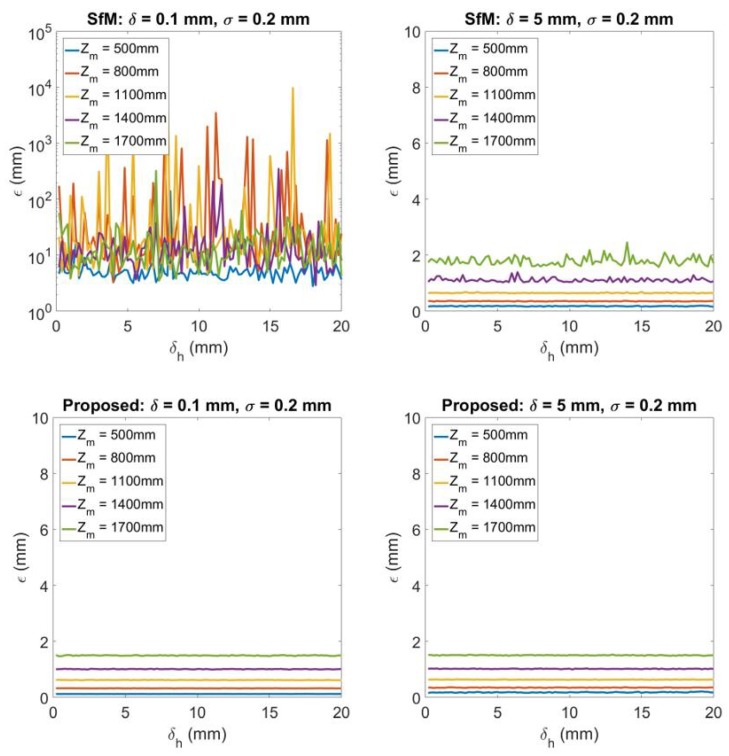
3D reconstruction error of different error $\delta_{h}=0.2,0.4,\ldots ,20$ for camera to road surface distance h. From left to right each column represents the results for δ=0.1,5 respectively. For each column, the top figure shows the 3D reconstruction by SfM, while the bottom figure illustrates 3D reconstruction by the proposed degenerate reconstruction technique.

Figure 11 shows the 3D reconstruction error when there exists a change of height $\delta_{h}$ caused by the vibration in camera to road surface distance *h*. The measured distance $\hat{h}$ is expressed as

(41) $$\begin{array}{c} \hat{h}=h-\Delta h\\ \Delta h∼U(0,\delta_{h}) \end{array}$$

where Δh is simulated to having a uniform distribution from 0 to $\delta_{h}$. In Figure 11 from left to right each column represents the result under δ=0.1,5 when σ is 0.2. Each top figure illustrates the results of using traditional SfM, while bottom figure represents the counterpart using the proposed technique. Withing each plot $\delta_{h}$ is changing from 0.2 to 20. The results show that when δ=0.1 the error from traditional SfM is large for the road surface. When δ=5 SfM starts to give comparable error with the proposed technique. For the proposed, it can be identified that when $\delta_{h}$ is changing from 0.2 to 20, the error remains almost the same for different $\delta_{h}$. It means that as the change of camera to ground height h is small during vehicle driving, it has little influence to the 3D reconstruction results by using the proposed technique.

**Figure 12:**
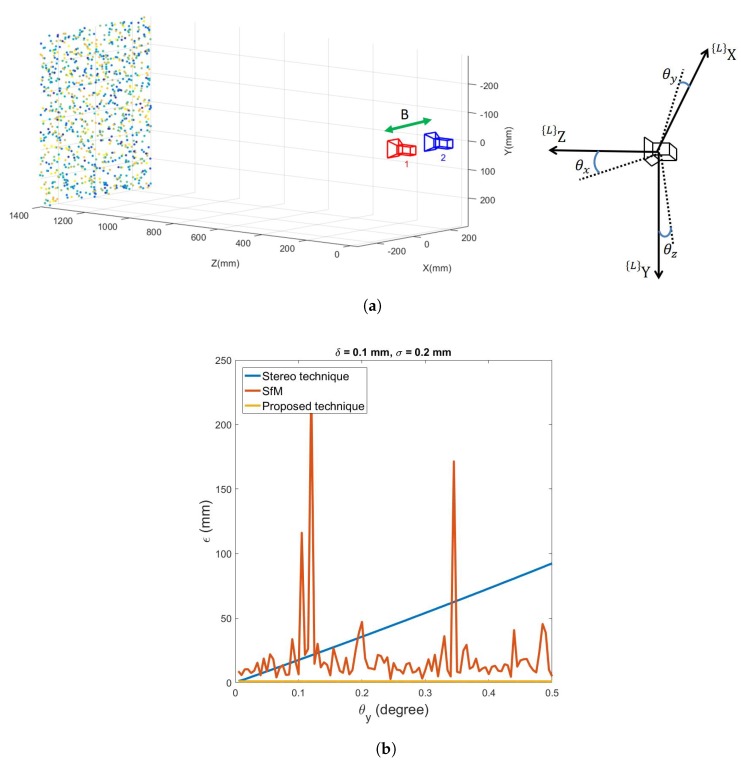
Comparison between stereo vision technique, traditional SfM, and the proposed technique under the influence of a changing *θ_y_* which is caused by the vibration. (**a**) Simulation environment for stereo vision-based technique. *B* is the baseline between the stereo cameras. (**b**) Reconstruction error for stereo technique, traditional SfM, and the proposed technique under vibration which causes changes to *θ_y_*.

Figure 12 illustrates the comparison between Fan’s [25] stereo vision road 3D reconstruction technique, traditional SfM, and the proposed technique. Figure 12a shows the simulation environment for stereo camera, where the baseline between the two cameras, *B*, is set to be B=200 mm. Figure 12b compares the 3D reconstruction error ϵ from a changing $\theta_{y}$, caused by the vibration of the vehicle, using stereo technique, traditional SfM, and the proposed technique on the same simulated road which has δ=0.1 mm and σ=0.2 mm. The camera(s) has a height h=1400 mm. To simplify the comparison, let $\theta_{y}$ be the angle for camera 2 respect to camera 1 caused by the vibration. It can be seen that the error from stereo technique exponentially increases when $\theta_{y}$ is larger. Even a relatively small vibration, when θ=0.1 degree, ϵ≈10 mm which is still large for the road surface reconstruction task. Although SfM has smaller error than the stereo technique most of the time after θ=0.2 degree, it still has a mean error which is over 10 mm. This is still mainly caused by the degenerate issue of the road surface 3D reconstruction. The proposed technique, however, has less than 2 mm reconstruction error which is mainly caused by the image noise σ.

### 4.2. Experiments on Real Road Surface

**Figure 13:**
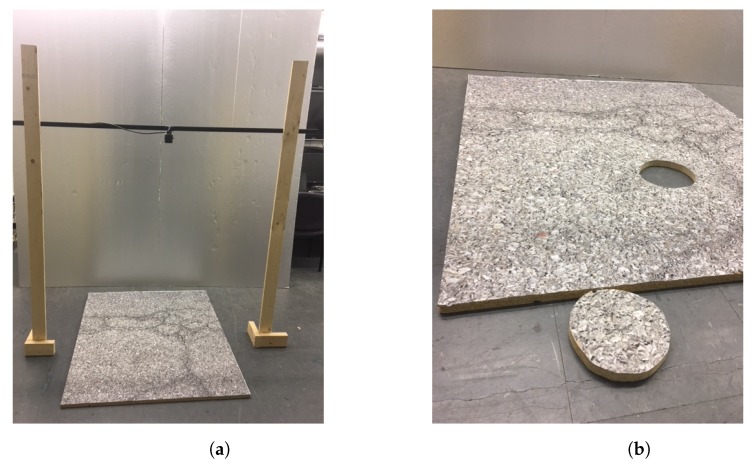
Experimental setup for accuracy analysis of the proposed 3D reconstruction technique. The camera to road distance *h* is set to be *h* = 900, 1000, 1100, 1200, 1300, 1400, 1500, 1600 mm. A flat plate with mimic road surface pattern is placed as a planar road. A hole on the plate can be used to simulate the road defect. (**a**) A height-adjustable gantry for the camera. (**b**) A flat plate sticked with a mimic road pattern image.

Figure 13 shows the experimental setup of the error analysis for the proposed technique using real images. The camera is facing downward to the road surface with its principle axis vertical to the ground surface as shown in Figure 13a. The ground surface is made by a flat plate to mimic a planar road surface as illustrated in Figure 13b. An image of road surface is printed and stuck to the flat plate to provide road surface patterns for the image feature searching and matching. A circular part of the plate can be removed from the plate to mimic the road pothole.

**Figure 14:**
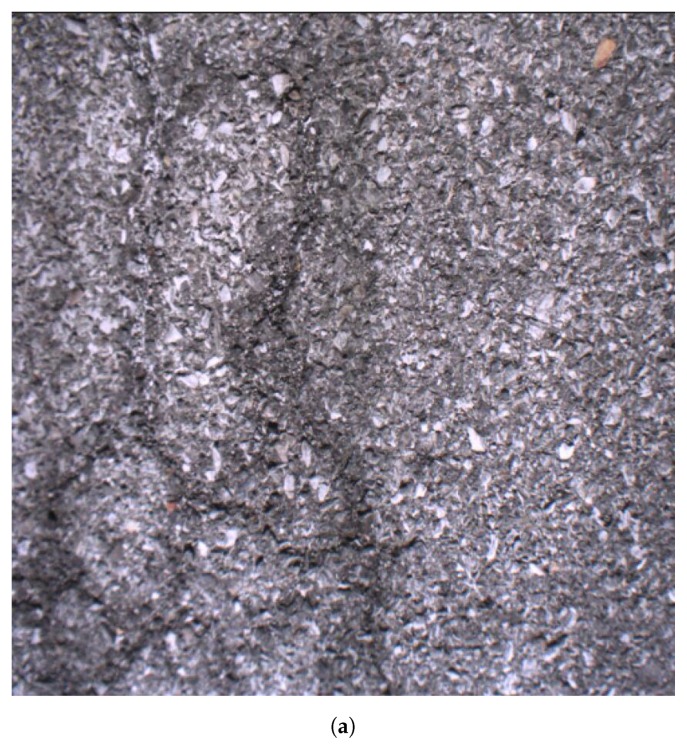

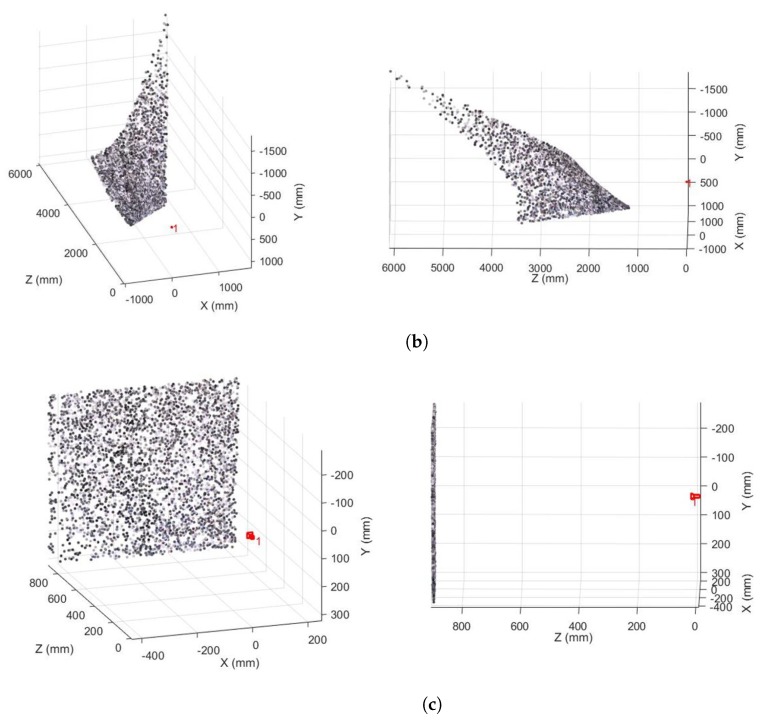
3D reconstruction of a flat plate using non-degenerate technique (SfM) and proposed degenerate technique. (**a**) An image of the flat plate sticked with mimic road pattern. (**b**) 3D reconstruction of flat surface in (a) using traditional SfM. The left image shows the front view of the reconstructed 3D points and the right image shows the left view. (**c**) 3D reconstruction of flat surface in (a) using the proposed technique. The left image shows the front view of the reconstructed 3D points and the right image shows the left view.

Figure 14 illustrates an example of the 3D reconstruction for a same flat plate image using SfM and the proposed technique. Traditional SfM fails in this example since the road surface in the image is a near-planar. However, the proposed one gives the correct planar-like 3D surface reconstruction as shown in Figure 14c.

**Figure 15:**
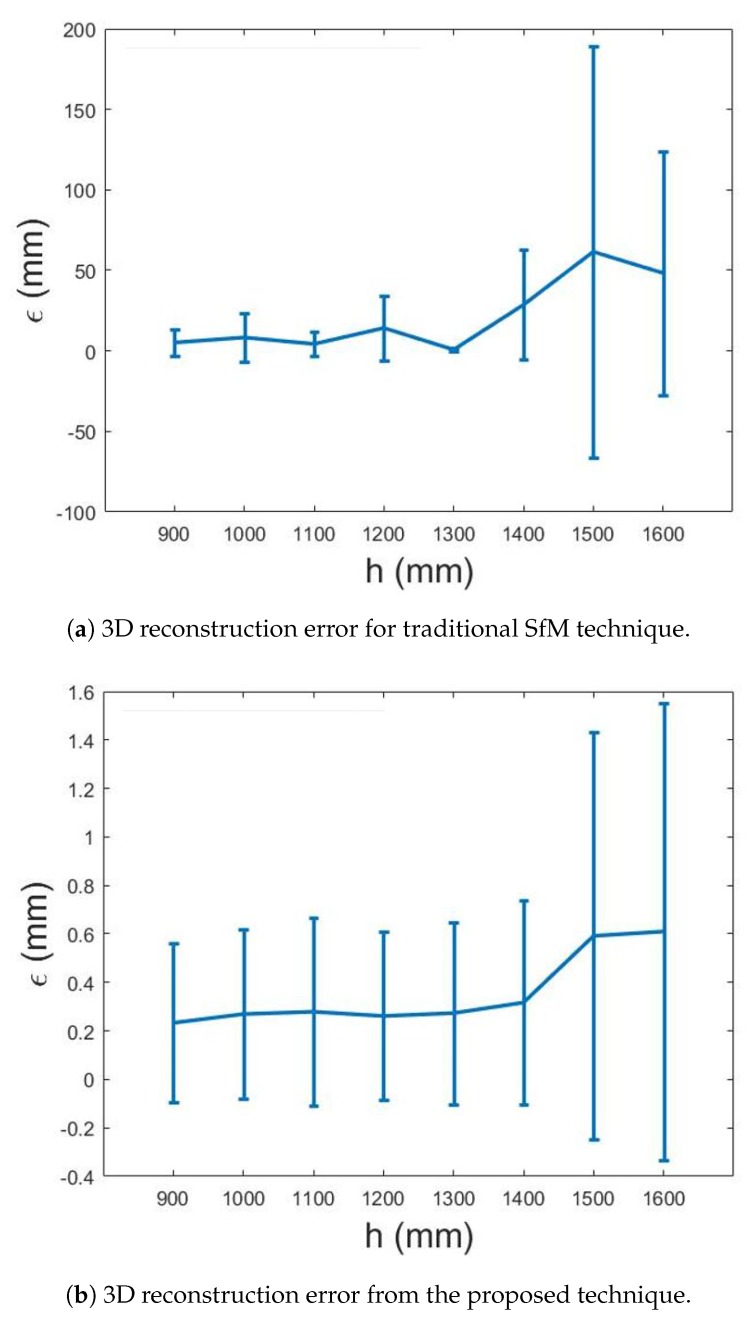
3D reconstruction error for traditional SfM technique and proposed technique at *h* = 900, 1000, 1100, 1200, 1300, 1400, 1500, 1600 mm.

Figure 15 shows the error analysis for 3D reconstruction using real images. Table 2 lists the parameters analyzed in the experiments using real images. It is noted that the mismatched feature rejection constant is found to be robust to keep correct matchings at λ=1.5. Figure 15a demonstrates the error of 3D reconstruction from traditional SfM. Figure 15b represents the 3D reconstruction error by using the proposed technique. The errors are compared between two techniques by changing the height of the camera *h* from 900 to 1600 mm. The mean errors are plotted and the error bar represents the standard deviation of 10 runs of image capturing for each height. It can be identified from Figure 15 that traditional SfM gives large mean error and standard deviation for this planar plate, while the proposed technique has mean error less than 0.6 mm and standard deviation close to 1 mm.

**Figure 16:**
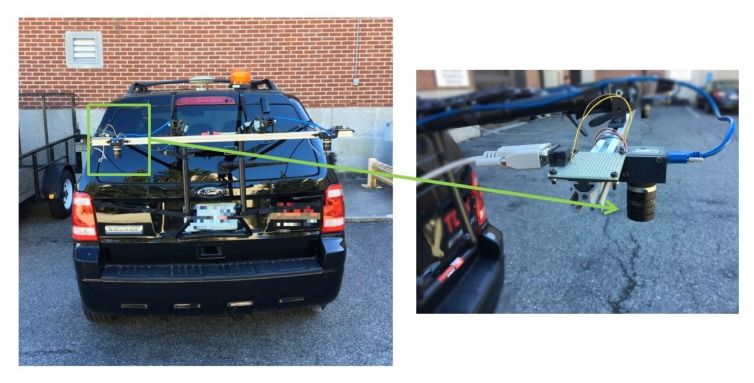
FPGA controlled road surface capturing system with adaptive camera frame rate.

Figure 16 shows a system which captures road surface images. The authors’ previous work [31] built this system which captures 1024×1280 resolution road surface images at a driving speed up to 100 km/hour. There are two cameras on this system. Although the proposed 3D reconstruction technique is based on a monocular camera, two cameras can work separately to increase the area of road surface region covered by images. This system is controlled by field-programmable gate array (FPGA) so that the frame rate of the camera is adaptive based on the vehicle speed. On-board diagnostics (OBD) port on the vehicle passes the vehicle’s velocity to FPGA which will set higher frame rate for the camera when the vehicle is moving fast and lower frame rate when the vehicle is slow. The system set the frame rate so that there is at least an 50% overlapping area between two consecutive images.

**Figure 17:**
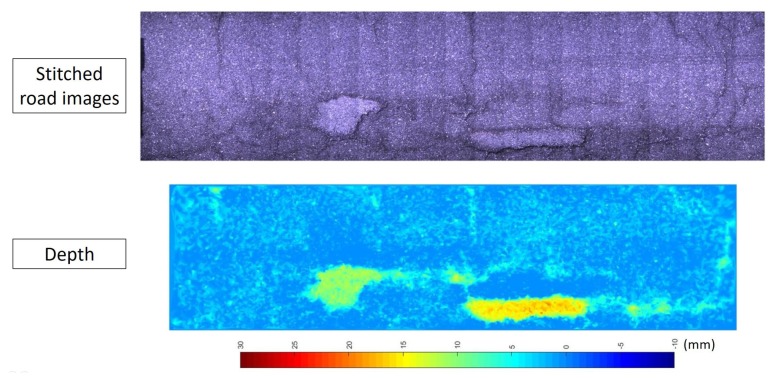
The qualitative 3D reconstruction result for a section of road using the proposed technique. Top: a road surface image stitched by 20 consecutive images captured. Bottom: A colormap image for the depth (^{G}^Z direction) values of reconstructed $X_{0:20}^{r}$ using the proposed techinque.

Figure 17 demonstrates the qualitative result of reconstructing the road surface using the proposed technique. The top figure shows a road surface image stitching for 20 images to visualize a section of road. The bottom one shows a colormap, which represents the depth (^{*G*}^Z direction) values of $X_{0:20}^{r}$ reconstructed by the proposed technique. It can be seen that two major defects together with several small defects are standing out from the 3D road surface.

**Figure 18:**
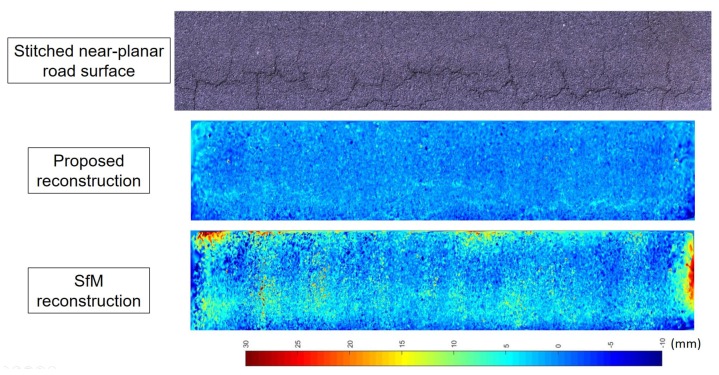
The comparison between the proposed and the traditional SfM technique for reconstructing a section of road surface. Top: a road surface image stitched by 20 consecutive images captured. Middle: A colormap image for the depth (^{*G*}^Z direction) values of reconstructed $X_{0:20}^{r}$ from the proposed techinque. Bottom: A colormap image for the depth ( ^{*G*}^Z direction) values of reconstructed $X_{0:20}^{r}$ using traditional SfM.

Figure 18 compares the proposed technique with traditional SfM in the quality of near-planar road surface reconstruction. The top figure is a near-planar road surface image stitched by 20 consecutive images. The middle one shows the colormap of the ^{*G*}^Z values of the reconstructed road $X_{0:20}^{r}$ for the same road using the proposed technique, while the bottom one is the reconstructed colormap of the same road obtained by using traditional SfM. It can be found that the proposed technique has much less outliers and noises in reconstructing the near-planar road surface. The proposed technique even differentiates small cracks by showing the different color at the crack areas. On the other hand, the same road surface reconstructed by the traditional SfM technique shows large deviated depth values at many places which are obviously not correct for a near-planar road surface.

**Figure 19:**
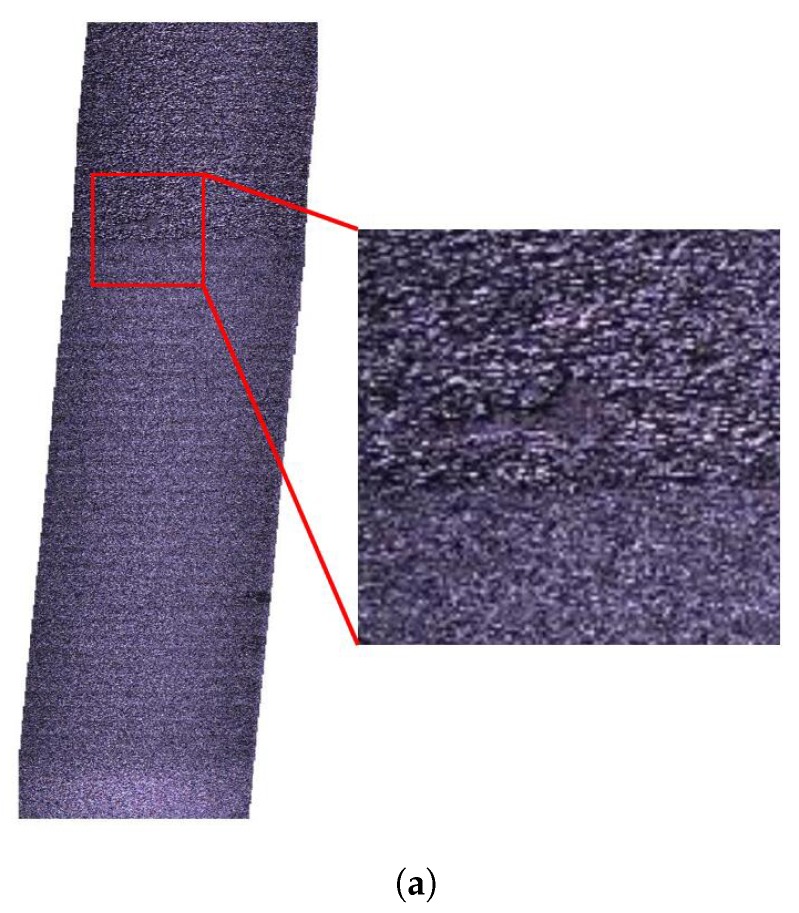

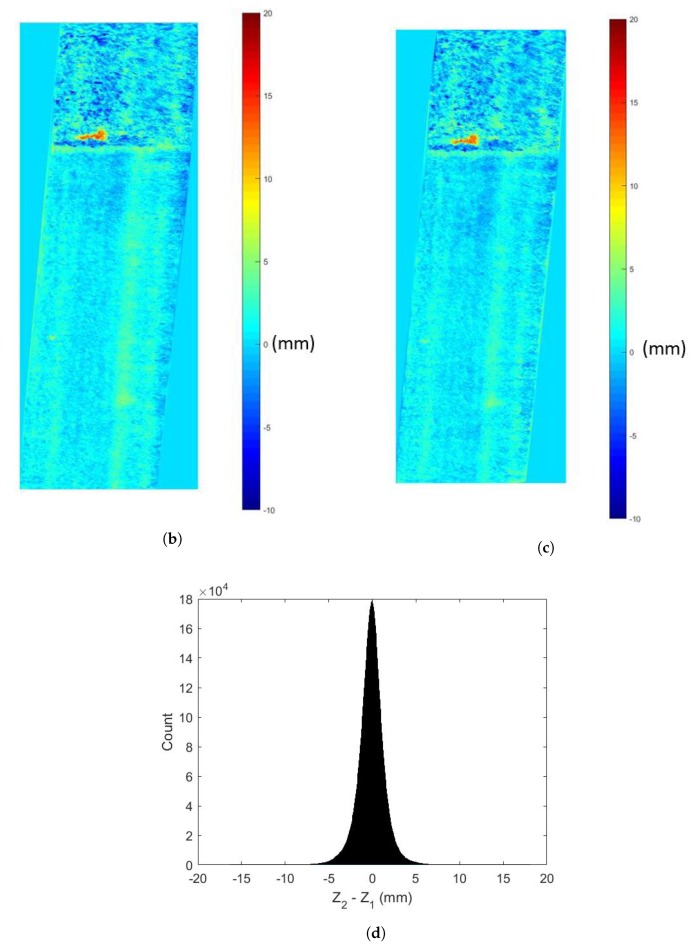
Repeatability test for the proposed technique. (**a**) Stitched images of a road section which has a geometrical defect. (**b**) The first measurement of one road section. ^{*G*}^$Z^{r}-h$ values of road surface point cloud data are represented by a colormap. (**c**) The second measurement of one road section. ^{*G*}^$Z^{r}-h$ values of road surface point cloud data are represented by a colormap. (**d**) Repeatability quantitative results. $Z_{1}$ are the ^{*G*}^$Z^{r}$ values from the first measurement while $Z_{2}$ are the ones from the second measurement.

Figure 19 demonstrates the repeatability experiment for the proposed technique. Figure 19a represents a section of the road which contains a pothole. This section of road surface are obtained by stitching 50 images which are captured using the system shown in Figure 16. In Figure 19b the ^{*G*}^$Z^{r}-h$ values of reconstructed 3D road surface points are plotted as a colormap. In Figure 19c, the proposed technique measures the same road section which is reconstructed in Figure 19b. The two measurements are then compared to validate the repeatability of the proposed technique. In Figure 19d, $Z_{1}$ are the ^{*G*}^$Z^{r}$ values of the reconstructed road surface points from the first measurement, while $Z_{2}$ are the ones from the second measurement. The histogram shows the count of $Z_{2}-Z_{1}$ values. The mean value of $Z_{2}-Z_{1}$ is −0.1079 mm and the standard deviation of $Z_{2}-Z_{1}$ is 1.3515 mm. The statistics results of $Z_{2}-Z_{1}$ reflects the high repeatability of the proposed technique.

Table 3 compares SfM with the proposed technique on defects detection using road surface images. The comparison is based on 6300 road surface images which are collected at rural, urban, and highway roads for weather conditions such as sunny, cloudy, and partly cloudy around Blackburg, Virginia area. The real road surface images are captured at both highway driving speed (100 km/h) and local road driving speed (40 km/h). Some images capture potholes while other images capture flat road surface. From true positive (TP), false positive (FP), true negative (TN), and false negative (FN), the accuracy is expressed as (TP+TN)/(TP+TN+FP+FN), precision as TP/(TP+FP), while the recall illustrated by TP/(TP+FN). From Table 3 although traditional SfM gives higher recall rate between the proposed technique and traditional SfM, it has only 34.34% precision rate. It means that although traditional SfM rarely misses the detection of potholes (less FN), it generates more wrong detection of potholes (more FP). The proposed technique on the other hand, results in 98.95% accuracy, 94.33% precision and 95.76% recall rate. All the three criteria are above 94%.

## 5. Conclusions

A geometry-based technique of reconstructing degenerate near-planar road surfaces from two images for road defects detection is presented in this paper. The proposed technique mathematically formulates the near-planar road surface reconstruction problem, and improves traditional SfM for the 3D road reconstruction process. Since the degenerate issue of the near-planar road surface reconstruction is solved by the proposed technique, road surface defects are thus detected from the accuracy-enhanced 3D road surfaces.

Two types of experiment were conducted to evaluate the proposed road surface 3D reconstruction for the defects detection technique. In the simulation environment, the first experiment compared SfM and the proposed technique under different road unevenness δ and the noise σ in images. Results showed that the changing of δ does not affect the reconstruction error ϵ using the proposed technique but increases ϵ dramatically for traditional SfM when δ is close to 0. The second experiment compared traditional SfM and the proposed technique under the different rotation angles $\theta_{x},\theta_{y},\theta_{z}$ for the camera. Results showed that by changing $\theta_{x},\theta_{y},$ and $\theta_{z}$ the error ϵ is less than 3 mm even at the worst case. The third experiment showed the change of camera to road distance $\delta_{h}$ almost does not change the ϵ when $0<\delta_{h}<20$ mm. The comparison of the stereo vision technique, traditional SfM, and the proposed technique demonstrated the robustness of the proposed technique for road surface reconstruction under the influence of vibration. For experiments using real images, the first experiment showed the 3D reconstruction error ϵ using both traditional SfM and the proposed technique for the reconstruction of a flat surface under laboratory environment. The results showed that the error for traditional SfM is much higher than the proposed technique, and the proposed technique has a mean error within 1 mm and standard deviation within 1 mm for h from 900 to 1600 mm. Lastly, 6300 real road surface images were captured by the presented system on both local road and highway road surfaces. The proposed technique increased the accuracy from 80% to 98.95% and precision from 34.34% to 94.33% for road defects detection.

This paper focused on reconstructing a 3D structure for road defects using a downward-facing camera. Future works include: 1. Making the camera facing forward to capture the images in front of the vehicle, and then detect defects and objects on the road surface to help vehicles avoid obstacles. 2. Using deep neural networks on both the images and 3D reconstructed points to improve the accuracy of road surface defects detection.

## Figures and Tables

**Table 1:** Parameters for simulated road surface and simulated camera.

Parameter	Value
Road unevenness: δ [mm]	0.1, 5, 10
Image noise: σ [pixel]	0.001, 0.002, …, 0.1
$Z_{m}$ [mm]	500, 800, 1100, 1400, 1700
$\theta_{x}$ [degree]	0.05, 0.10, …, 5
$\theta_{y}$ [degree]	0.05, 0.10, …, 5
$\theta_{z}$ [degree]	0.05, 0.10, …, 5
Two-view translation
**t** [mm, mm, mm]	${\left(200,30,0\right)}^{T}$
Change of h: $\delta_{h}$ [mm]	0.2, 0.4, …,20

**Table 2:** Parameters for experiments using real images.

Parameter	Value
Camera Field of View	$56^{\circ }\times 44^{\circ }$
Road unevenness: δ [mm]	<0.5
Camera to road distance: h [mm]	900, 1000, …,1600
Image noise: σ [pixel]	<0.2
Mismatched feature rejection constant: λ	1.5
Two-view camera translation:
**t** [mm, mm, mm]	${\left(100,0,0\right)}^{T}$

**Table 3:** Performance of road surface defects detection for different techniques.

	Proposed	SfM
TP	632	658
TN	5602	4382
FP	38	1258
FN	28	2
Accuracy	98.95%	80%
Precision	94.33%	34.34%
Recall	95.76%	99.70%

## Appendix A

To solve $F_{k}$, Equation (5) is rearranged to a form of $Af_{k}=0$, where:

(A1) $$\begin{array}{c} A_{i}=\left[\begin{array}{c} x_{k,i}^{r}x_{k-1,i}^{r}\\ x_{k,i}^{r}y_{k-1,i}^{r}\\ x_{k,i}^{r}\\ y_{k,i}^{r}x_{k-1,i}^{r}\\ y_{k,i}^{r}y_{k-1,i}^{r}\\ y_{k,i}^{r}\\ x_{k-1,i}^{r}\\ y_{k-1,i}^{r}\\ 1 \end{array}\right],\;A=\left[\begin{array}{c} A_{1}^{T}\\ A_{2}^{T}\\ .\\ .\\ .\\ A_{n}^{T} \end{array}\right]\\ f_{k}={\left(f_{11},f_{12},f_{13},f_{21},f_{22},f_{23},f_{31},f_{32},f_{33}\right)}^{T} \end{array}$$

In SfM [30], $F_{k}$ is obtained by solving the minimization problem:

(A2) $$\min_{f_{k}}Af_{k}$$

subject to

(A3) $$∥f_{k}∥=1$$

After the fundamental matrix $F_{k}$ is calculated, by following the subsequent SfM process, the essential matrix $E_{k}$ is calculated as:

(A4) $$E_{k}=K^{T}F_{k}K$$

where **K** is the intrinsic matrix of the calibrated camera. The Singular Value Decomposition (SVD) of $E_{k}$ then contributes to the calculation of rotation matrix $R_{k}$ and the up-to-scale translation vector $t_{k}$ between time step k−1 and *k*:

(A5) $$\begin{array}{c} E_{k}=UDV^{T}\\ W=\left[\begin{array}{ccc} 0 & -1 & 0\\ 1 & 0 & 0\\ 0 & 0 & 1 \end{array}\right]\\ R_{k}=UWV^{T}\;or\;R_{k}=UW^{T}V^{T}\\ t_{k}=U{\left(0,0,1\right)}^{T}\;or\;t_{k}=-U{\left(0,0,1\right)}^{T} \end{array}$$

where there is one correct combination of $R_{k}$ and $t_{k}$ which can make all $X_{k}^{r}$ be in front of the camera. The projection matrix $P_{k}$ is identified by the rotation matrix $R_{k}$ and the translation vector $t_{k}$. The 3D reconstructed points $X_{k}^{r}$ are finally obtained by the triangulation $f_{t}\left(\cdot \right)$:

(A6) $$\begin{array}{c} x_{k-1}^{r}=P_{k-1}X_{k}^{r}=K\left[I,0\right]X_{k}^{r}\\ x_{k}^{r}=P_{k}X_{k}^{r}=K\left[R_{k},t_{k}\right]X_{k}^{r} \end{array}$$

(A7) $$X_{k}^{r}=f_{t}\left(K,R_{k},t_{k},x_{k}^{r},x_{k-1}^{r}\right)$$
